# Is nutritional status associated with depression? evidence from a cross-sectional study among workers in tertiary educational institutions in Southwestern Nigeria

**DOI:** 10.11604/pamj.2021.39.94.23567

**Published:** 2021-06-02

**Authors:** Adeleye Adeomi, Chukwubueze Obiajunwa, Olajuwon Oduntan, Ebuka Ogbukwo

**Affiliations:** 1 Community Health Department, Obafemi Awolowo University, College of Health Sciences, Ile-Ife, Osun State, Nigeria

**Keywords:** Depression, nutritional status, tertiary, mental health, educational institution, underweight, overweight, obesity

## Abstract

**Introduction:**

different studies have shown a relationship between depression and nutrition, but there seems to be no consistent consensus on this. This study therefore investigated the relationship of nutrition status and depression among workers in tertiary educational institutions in Southwestern Nigeria.

**Methods:**

this was a cross-sectional study conducted among 399 members of staff of three tertiary educational institutions in Osun State, Southwestern Nigeria. Depression was assessed using the Patient Health Questionnaire-9 (PHQ-9), while nutritional status was assessed using the body mass index (BMI), waist circumference and waist hip ratio (WHR). The respondents were selected using multi-stage sampling technique, and data were collected using pre-tested structured questionnaires. Analysis was done using IBM SPSS.

**Results:**

the mean age of the respondents was 45.8 ± 10.4 years. The prevalence of depression was 23.8%. Concerning the nutritional status of respondents, 2.3% were underweight and 69.7% were overweight/obese. There were statistically significant associations between depression and the nutritional status of the respondents using BMI (p = 0.001), WHR (p = 0.015) and waist circumference (p = 0.036). After controlling for other factors, only the BMI was still significantly associated with depression, such that those underweight were more likely to be depressed (Odds ratio: 7.9; p-value: 0.009).

**Conclusion:**

the prevalence of depression among the respondents was relatively high, and this was significantly associated with the BMI, even after controlling for co-founders.

## Introduction

Depression is a common mental disorder globally, and Nigeria is not an exception. The World Health Organization (WHO) reports that more than 300 million people (4.4%) are affected globally, with an 18.4% increase in prevalence from 2005 to 2015 [1]. The prevalence of depression in Nigeria is 3.9%, which in actual numbers means that more than 7 million Nigerians have depressive disorders [1]. The increasing prevalence of suicide in Nigeria has further necessitated more interest in depression and the possible ways to prevent or manage it [2]. Different studies have shown a relationship between depression and nutrition, but there seems to be no consistent consensus on the pattern of relationship. While different studies have shown that depression significantly increased with under-nutrition [3-5], other studies have reported a significant increase in depression with over-nutrition, in the form of abdominal or generalized obesity [6]. Establishing the presence and direction of association between depression and nutrition is important, especially because of the potential it holds for the control and prevention of depression. Nutrition, being largely modifiable holds a great potential for the prevention and management of depression if the relationship is well understood. This study therefore aimed to assess the relationship between depression and the nutritional status among the staff members of tertiary educational institutions in Southwestern Nigeria.

## Methods

**Study location and study population:** the study was carried out among three tertiary educational institutions in Osun State, Southwestern Nigeria. These were randomly picked from the list of all the tertiary educational institutions in Osun State using simple random sampling technique (balloting method). The respondents were adults and had been in the employment of the institutions for a minimum of 2 years prior to data collection. Those who were acutely ill or had chronic illnesses like sickle cell disease anaemia that could affect their weight were excluded. Others with disabilities that made them unable to stand were also excluded.

**Sample size and sampling technique:** the sample size was calculated to get an absolute precision of ± 5% using STATCALC on the Epi-Info software. The proportion of expected outcome was taken as 38.3% which was the proportion of obese staff in a tertiary educational institution also in Southwestern Nigeria [7]. After correcting for an anticipated 10% non-response, a total of 399respondents were recruited using multi-stage sampling technique.

**Research instruments and data collection methods:** depression was assessed using the Patient Health Questionnaire-9 (PHQ-9), which both diagnoses and assesses the severity of depression, and has been widely used for similar studies. Height was measured to the nearest 0.1 meter using the stadiometer (Leceister^®^Height Measure, Seca, UK), weight was measured using the Seca® electronic bathroom weighing scale (SECA GmbH & Co, Germany) and waist and hip circumferences using the Goldfish brand non-elastic tape measure. The anthropometric measurements were done according to standard protocols recommended by the International Society for the Advancement of Kinanthropometry [8].

**Measurement of outcome variables:** depression was assessed using the Nine questions as contained in PHQ-9, and responses were scored 0 (not at all) to 3 (nearly every day), giving a minimum score of 0 and a maximum score of 27. The total score for each respondent was then used to categorize them into Minimal or no depression (0-4), mild depression (5-9), moderate depression (10-14), moderately severe depression (15-19) and severe depression (20-27). Nutritional status of the respondents was determined using the Body Mass index (BMI), which was calculated by dividing the weight in kilograms by the height in meter^2^. The BMI was then used to categorize respondents into underweight (< 18.5), normal weight (18.5-24.9), overweight (25-29.9) and obese (30 and above). Nutritional status was also assessed using the waist circumference (WC) and waist-hip ratio (WHR), according to the WHO classifications. Men and Women with WC ≥ 94cm and ≥ 80cm, and those with WHR ≥ 0.90 and ≥ 0.85 were at risk of metabolic diseases respectively.

**Data analysis**: the questionnaires were manually sorted out, entered into a computer and the obtained data was analyzed using IBM SPSS version 25. Descriptive analysis of all the variables measured were first done, and the categorical variables were reported as frequencies and proportions/percentages, while the continuous variables were reported as means ± standard deviation. Cross-tabulations were done to test for associations between the different categorical variables (in line with the objective of the study) using the chi-square test. Two independent samples t-test was used to compare the means of ages, dietary diversity scores and waist circumference between those with depression and those with no/minimal depression. Binary logistic regression was done to identify the factors associated with depression. The age, sex and other factors with p-value < 0.25 were entered into the model. Level of significance was set at p < 0.05 for this study.

**Ethical considerations:** ethical clearance was obtained from the Ethical Review Committee, Institute of Public Health, Obafemi Awolowo University, Ile-Ife. The participants´ information sheet and consent were given to the respondents. Important information on the participants´ information sheet included that the information volunteered will be kept confidential as all questionnaires were coded without names or addresses of respondents. It also emphasized that participants were free to opt-out if they were not comfortable with the information in the questionnaire. Signed consent forms were then obtained from the respondents before they were included in the study.

## Results

**Figure 1:**
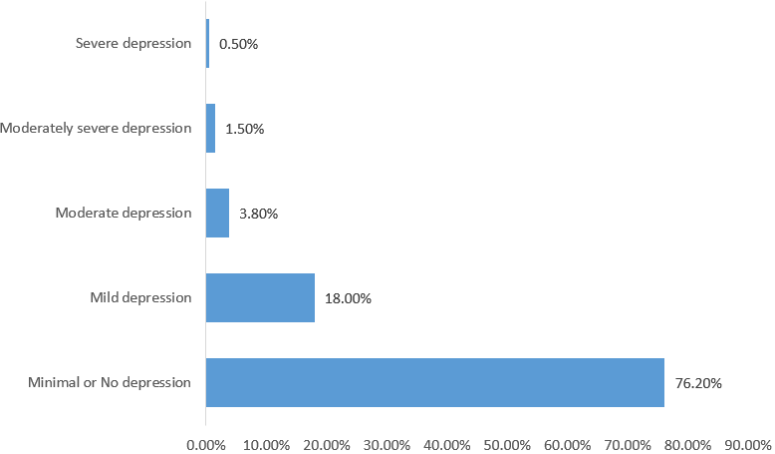
depression severity categories among the respondents (N = 399)

The mean age of the respondents was 45.8 ± 10.4 years, more of them were females (51.6%) and 344 (88.2%) were Yoruba by tribe. Most of the respondents were married (87.7%), and were from monogamous family settings (92.0%). Concerning their job designation, majority were non-academic staff (60.9%). One hundred and eight-six (46.6%) of the respondents earned below 100,000 naira ($278) monthly, while the remaining earned more. Concerning the nutritional status of the respondents, using BMI, 2.3% were underweight, 43.9% were overweight, 25.8% were obese, and others were normal. Using WC and WHR, 54.6% and 60.2% were at risk of metabolic diseases. The symptoms of depression as assessed using the PHQ-9 is shown in Table 1, the distribution of respondents according to depression categories is shown in Figure 1. Overall, the prevalence of depression was 23.8% among the respondents, most (75.8%) of which had mild depression.

**Table 1:** depressive symptoms among respondents (N = 399)

Mood symptoms	Frequency	Percentage (%)
**Little interest**		
Not at all	291	72.9
Several days	79	19.8
More than half the days	16	4.0
Nearly every day	13	3.3
**Feeling down**		
Not at all	292	73.2
Several days	85	21.3
More than half the days	16	4.0
Nearly every day	6	1.5
**Sleep disturbance**		
Not at all	275	68.9
Several days	87	21.8
More than half the days	28	7.0
Nearly every day	9	2.3
**Reduced energy**		
Not at all	232	58.1
Several days	118	29.6
More than half the days	32	8.0
Nearly every day	17	4.3
**Appetite changes**		
Not at all	324	81.2
Several days	54	13.5
More than half the days	14	3.5
Nearly every day	7	1.8
**Feeling bad**		
Not at all	329	82.5
Several days	55	13.8
More than half the days	9	2.3
Nearly every day	6	1.5
**Trouble concentrating**		
Not at all	299	74.9
Several days	76	19.0
More than half the days	18	4.5
Nearly every day	6	1.5
**Moving slowly**		
Not at all	361	90.5
Several days	22	5.5
More than half the days	12	3.0
Nearly every day	4	1.0
**Suicidality**		
Not at all	379	95.0
Several days	15	3.8
More than half the days	4	1.0
Nearly every day	1	0.3

Among the socio-demographic characteristics, the age and educational status were significantly associated with depression, such that the younger respondents and the less educated were more likely to be depressed compared with the others (Table 2). These relationships were still statistically significant even after the variables were entered into the logistic regression model (Table 3). The nutritional status of the respondents as measured using BMI was significantly associated with depression, such that those underweight were more likely to be depressed (Table 2). The relationship was still statistically significant after controlling for socio-demographic characteristics using logistic regression. Underweight respondents were 8 times more likely to be depressed than overweight/obese respondents. The waist circumference and waist-hip ratio were significantly associated with depression at the bivariate level (Table 2), but were no longer significant after controlling for confounders (Table 3).

**Table 2:** relationship between selected socio-demographic characteristics, nutritional status and depression among the respondents

Variable	Depression Categories (%)		Statistics
	**No depression**	**Depression**	
**Age in years**	46.9 ± 10.3	42.3 ± 9.9	ϯp< 0.001*
**Gender**			b^2^ = 2.023, df = 1, p = 155
Male	141(73.1)	52(26.9)	
Female	163(79.1)	43(20.9)	
**Highest level of education**			^2^ = 10.642, df = 4, p = 0.031*
Secondary	55(71.4)	22(28.6)	
Bachelors	37 (86.0)	6(14.0)	
Masters	48(71.6)	19(28.4)	
Doctorate	86(85.1)	15(14.9)	
Others	78(70.3)	33(29.7)	
**Marital status**			^2^ = 2.443, df = 2, p = 0.295
Single	24(66.7)	12(33.3)	
Married	271(77.4)	79(22.6)	
Widowed	9(69.2)	4(30.8)	
**Monthly income**			^2^ = 7.431, df = 4, p = 0.115
Less than ₦50,000	81 (73.6)	29 (26.4)	
₦50,000 - ₦99,000	53 (69.7)	23(30.3)	
₦100,000-₦249,000	92(76.0)	29(24.0)	
₦250,000-₦500,000	45(80.4)	11(19.6)	
Greater than ₦500,000	33(91.7)	3(8.3)	
**Body Mass Index**			LR = 13.692, df = 2, p = 0.001*
Underweight	3 (33.3)	6 (66.7)	
Normal	77(68.8)	35(31.2)	
Overweight/Obese	224(80.6)	54 (19.4)	
**Waist circumference**			^2^ = 4.420, df = 1, p = 0.036*
Normal	129(71.3)	52(28.7)	
At risk	175(80.3)	43 (19.7)	
**Waist-hip ratio**			^2^ = 5.930, df = 1, p = 0.015*
Normal	111(69.8)	48(30.2)	
At risk	193(80.4)	47(19.6)	

* - Statistically significant ϯ2-independent sample-test used, ^2^ - chi-square test LR - Likelihood ratio

**Table 3:** determinants of depression using binary logistics regression analysis

Variables	Odds ratio	Confidence interval	p-value
**Age in years**	0.963	0.934-0.994	0.019*
**Gender**			
Male	1.450	0.777-2.704	0.243
Femaleϯ			
**Occupation**			
Academic staff	1.831	0.508 - 6.593	0.355
Non-academic staffϯ			
**Highest level of education**			0.119
Secondary	1.469	0.674-3.197	0.332
Bachelors	0.287	0.097 - 0.849	0.024*
Masters	0.433	0.113 - 1.662	0.223
Doctorate	0.313	0.061 - 1.611	0.165
Othersϯ			
**Monthly income**			0.491
Less than ₦50,000	0.980	0.169-5.665	0.982
₦50,000-₦99,000	1.746	0.329 - 9.258	0.513
₦100,000 - ₦249,000	1.744	0.392 - 7.752	0.465
₦250,000 - ₦500,000	2.111	0.519-8.582	0.296
Greater than ₦500,000ϯ			
**Body Mass Index**			0.020*
Underweight	7.867	1.684-36.747	0.009*
Normal	1.539	0.873 - 2.712	0.136
Overweight/Obeseϯ			
**Waist circumference**			
Normal	0.750	0.382 - 1.470	0.401
At riskϯ			
**Waist-hip ratio**			
Normal	1.373	0.799 - 2.357	0.251
At riskϯ			

* - Statistically significant ϯReference variable

## Discussion

The prevalence of obesity was disturbingly high among the respondents with nearly 7 out of 10 of the respondents being overweight/obese. Using waist circumference and waist-hip ratio, about three-fifths of the respondents had abnormal values and were at risk of metabolic diseases. Similar studies that have been carried out among workers in tertiary educational institutions in Nigeria have reported prevalence rates in excess of 60% for overweight and obesity [9-11]. This pattern is disturbing, because it is in tertiary educational centres that researches that drive all aspects of the National life take place, and therefore would require healthy workers. This high level of overweight/obesity with its attendant cardiovascular risk should therefore be a source of concern to all stakeholders.

Using the PHQ-9, a fifth of the respondents had mild to severe depression. Majority of these (18%) had mild depression, while less than 1% (0.5%) had severe depression. Although no other study on depression among tertiary educational institutions workers could be found in Nigeria as at the time of writing this, the prevalence of depression (21.4%) reported by a study among the elderly in Southwestern Nigeria were similar to what was found in this study [12]. Another study among adults attending an out-patient clinic in Southwestern Nigeria reported a prevalence of 59.6% [13], while a study carried out among adults in Oyo State reported a prevalence rate of 5.3% for depression [14]. Comparing the prevalence of depression determined during surveys of this nature could be challenging because of the differing tools which sometimes make comparison difficult. The study in Oyo state for instance, first used the general health questionnaire (GHQ 12) to screen the respondents and proceeded at the second phase to use the Structured Clinical Interview DSM IV for assessment of clinical depression [14]. The other studies, including this study, however only used screening tools at the population level.

Depression was significantly associated with the nutritional status using BMI, waist circumference and waist-hip ratio, such that those who had generalized overweight/obesity, abdominal obesity and raised waist-hip ratio were less likely to be depressed. After controlling for socio-demographic confounders, there was still a significant relationship between nutritional status measured using BMI and depression, such that those underweight were nearly 8 times more likely to be depressed than those overweight/obese. This finding corroborates the finding of some studies [3-5, 15], that depression was significantly higher among underweight adults. A large study among overweight and obese adults in United States of America however found that abdominal obesity was significantly associated with increased prevalence of depressive disorders [6]. Another large study reported that depression was significantly higher among those with elevated WHR [16].

The reason for this inconsistency in the relationship between depression and nutritional status is not clearly known. The differences in study designs, sample sizes, instruments for assessing depression and assessment of nutritional status as used by the different studies may be responsible for the inconsistency. It is also worthy of note that the studies reporting association between obesity and increased prevalence of depression are from western countries, while most studies in the developing countries report an inverse relationship between obesity and depression. There may be need for further studies to clarify these. A limitation of the present study is the study design (i.e. cross-sectional) which limits causal inferences. Another limitation is that the diagnosis of depression using PHQ-9 may not correspond with the clinical diagnosis of depression.

## Conclusion

The prevalence of depression among the respondents was relatively high, and this was significantly associated with the BMI, even after controlling for co-founders. It was such that those underweight were 8 times more likely to be depressed than those overweight/obese. There is need for more interest and intervention by stakeholders into the mental and nutritional health of tertiary educational institution workers in Nigeria.

### What is known about this topic

- Depression is a common mental disorder globally, and Nigeria is not an exception;
- The prevalence of depression in Nigeria is 3.9%;
- Different studies have shown a relationship between depression and nutrition, but no consensus on the pattern of relationship.

### What this study adds

- The prevalence of depression among workers in tertiary educational institutions in Southwestern Nigeria was 23.8%;
- The nutritional status of the respondents as measured using BMI was significantly associated with depression, such that those underweight were more likely to be depressed.
